# A Pancreatic Fistula after the Laparoscopic Sleeve Gastrectomy

**DOI:** 10.1155/2015/910583

**Published:** 2015-05-11

**Authors:** Gökhan Selçuk Özbalcı, Ayfer Kamalı Polat, İsmail Alper Tarım, Murat Derebey, Mehmet Selim Nural, Volkan Tümentemur, Ufuk Karabacak

**Affiliations:** ^1^Department of General Surgery, Faculty of Medicine, Ondokuz Mayıs University, 55139 Samsun, Turkey; ^2^Department of General Surgery, Varto State Hospital, 49100 Muş, Turkey; ^3^Department of Radiology, Faculty of Medicine, Ondokuz Mayıs University, 55139 Samsun, Turkey

## Abstract

Laparoscopic sleeve gastrectomy (LSG) is a popular surgical weight-loss procedure in the treatment of morbid obesity. There are some complications regarding this procedure in the literature. This report presents a pancreatic fistula (PF) case, which has not been previously seen.

## 1. Background

Laparoscopic sleeve gastrectomy (LSG) has recently become a popular method of surgical treatment for morbid obesity [1]. This technique is superior due to the fact that it does not disturb the integrity of the gastrointestinal system; it contains no external material and has low complication ratios [2]. The most common complications of LSG are leakage (2.2%) and suture line bleeding [3, 4].

Pancreatic fistula (PF), post-LSG, so far has not been reported. PF mostly develops after pancreatic disorders or as a result of trauma, such as pancreatic surgery, abdominal trauma, or percutaneous radiologic procedures. In addition, it has been reported that pancreatic fistula may develop after surgery involving adjacent organs [5]. We, however, report a post-LSG pancreatic fistula case, which does not yet exist in the literature.

## 2. Case Presentation

A 43-year-old morbidly obese man underwent LSG in another hospital 32 days prior to admittance to our hospital. Patient's height was 179 centimeters, body weight was 160 kilograms, and body mass index (BMI) was 50 kg/m^2^. There were no additional disease and no comorbidity. After the sixth day of his operation, the patient was suffering from 39°C fever and a spit-like accumulation of 100 mL/day from the 22 French silicon drain located near his stomach. The initial suspicion was that there could be a stapler line leakage. Therefore, the patient was not discharged and was observed further in the following 25 days. Fluid accumulation in the patient's drain did not decrease. An endoscopic stent was applied twice; however, the procedures were a failure.

After this experience, the patient demanded to be discharged from the hospital and admitted to our institution. He did not have oral intake. His initial fever was 36.9°C, his tension was 120/80, his pulse was 88 beats/min, his Hb was 15.1 g/dL, his WBC was 8.27/mm^3^, his Plt was 331/mm^3^, his amylase was 460 U/L, and his lipase was 208 U/L. Initially, a leakage test using methylene blue was conducted on the patient and no fluid in the drain was observed. Then, oral-intravenous contrasted computer tomography (CT) was performed. The drain was seen along the stapler line and there was no leakage on the CT examination.

**Figure 1:**
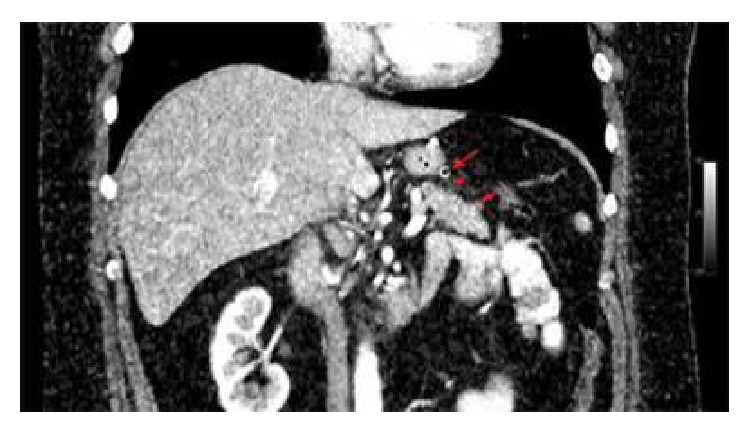
Long arrow: the drain; short arrow: pancreatic corpus; arrow head: focal contamination on the fatty tissue between the pancreas and drain.

A minimal pollution was observed between the stomach and pancreas. It was considered a postoperative change and a pathological case was not considered (Figure 1). Meanwhile, 30–100 mL/day fluid was continuously discharged by the drain. On the fifth day of our clinical follow up, a new oral contrasted dynamic CT was performed due to the suggestion from the radiology department. There was no leakage observed in that test either. As a result, it was suspected that a pancreatic fistula may have occurred in the patient. Thus, drain and spit fluid were sent to the laboratory and examined for amylase level. The spit fluid amylase level was 297.900 U/L and the drain amylase was 61.499 U/L. The drain amylase level led us to believe that there was a pancreatic fistula in the patient. Upon this observation, octreotide, a somatostatin analogue, was started with the subcutaneous dosage of 3 × 0.1 mg/mL [6]. The patient dramatically responded to the treatment. After 48 hours, fluid accumulation from the drain stopped and oral intake was started. The treatment continued in the following three days. Additionally, intravenous medication consists of 3500 cc/day total parenteral nutrition, 40 mg/day omeprazole, and 60 mEq/day potassium chloride, which was given until oral intake started. Also 7500 IU/0,3 mL/day bemiparin sodium was given as long as the patient was hospitalized. On the 12th day after the patient was admitted to our clinical, his blood test results were as follows: Hb: 12.7 g/dL, WBC: 5.9/mm^3^, Plt: 291/mm^3^, amylase: 138 U/L, and lipase: 107 U/L. The patient's drain was uninstalled on the 12th day and he was discharged on the 13th day. Further follow-ups and tests on the patient in the following year showed that he had no further complaints.

## 3. Discussion

It is estimated that there are over 300 million obese adults in the world [7]. Obesity should not be seen as only a cosmetic problem due to its association with increased risk of type II diabetes, hypertension, cardiovascular disease, dyslipidemia, nonalcoholic steatohepatitis, arthritis, choledolithiasis, sleep-apnea syndrome, and many types of cancer [8].

People with a BMI of 40 or greater, who have tried diet and exercise, who do not have an endocrine disease, who are not addicted to alcohol or drugs, and who are psychologically stable, are candidates for bariatric surgery. Additionally, patients with a BMI of 35 to 40 kg/m^2^ for weight-loss who are expected to improve type 2 diabetes, cardiorespiratory diseases, and obesity-related conditions, such as severe joint disease and serious psychological disorders, are suitable for bariatric surgery [9].

Weight-loss and metabolic effects in patients who have undergone LSG can be achieved by a reduction in the stomach volume capacity and thus a reduction in food intake, as well as mechanical and hormonal changes. LSG has also low mortality and morbidity, and its short-term results are very effective for appropriate patients with a BMI of 40 kg/m^2^ or greater who instituted but failed an adequate exercise and diet program. The most common complications are leakage and bleeding from the suture line [1–4]. However, a PF after LSG is a complication that has not been encountered in the literature. In our critical opinion, we suspected a minimal injury caused by anatomical closeness in our patient who was operated on in another hospital. The location of the drain on a major curvature through the pancreas provided drain leakage and avoided the development of peritonitis. Efficiently providing drainage prevented the radiologic observation of fluid accumulation around the pancreas. At the same time, although the patient had the operation long time ago and the drain index was not as expected, pancreatic markers were reasonably high.

## 4. Conclusion

**Figure 2:**
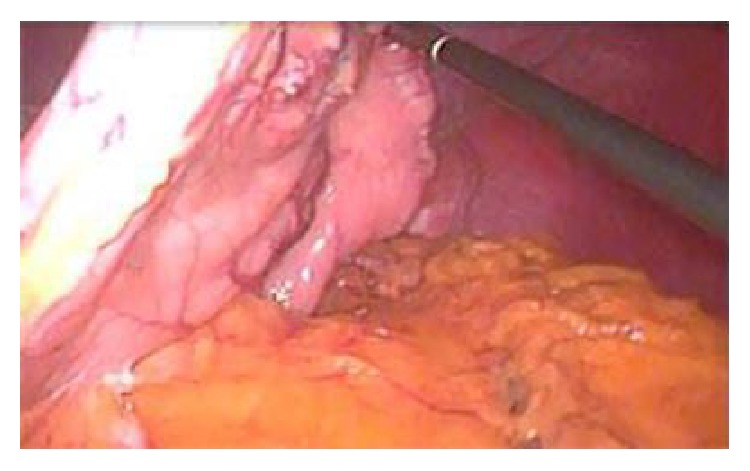
Elevation of the stomach.

**Figure 3:**
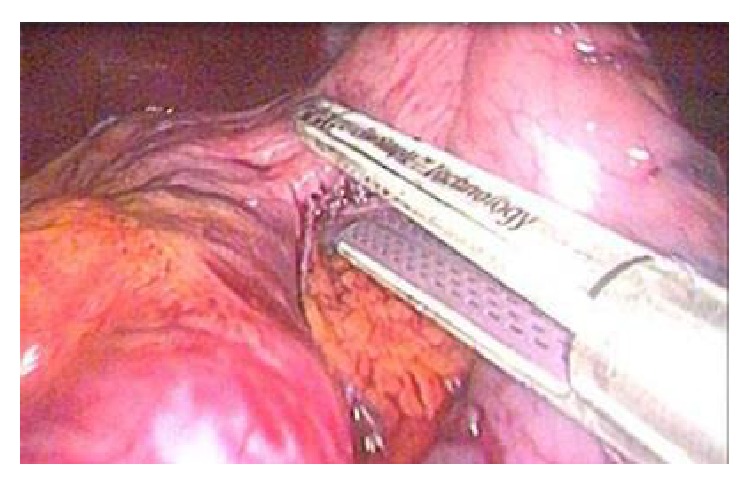
Placing stapler's blunt and thick side facing down.

LSG is an efficient surgical technique for patients suffering from morbid and super morbid obesity. There are a few important ways to avoid complications during LSG. The first one is to elevate the stomach during dissection with ligaSure and to check the posterior face in every state as shown in Figure 2. The second way is by placing the stapler's blunt and thick side facing down and there must be a clear vision of what is in the stapler branches. Thus, we believe that the surrounding solid organs and vessel structures will be protected (Figure 3). The last point is that, before the firing of the stapler, the stomach should be hanged and checked, and, thus, the lower leg of the stapler should not touch any organs. Additionally, we have to make sure that there is no tissue between the legs of the stapler in order for the operation to be conducted securely. However, despite the present case report, a PF is an extremely rare short-term side effect in patients with LGS.
